# Supplementary material for: Transcriptome Sequencing Reveals Pathways Related to Proliferation and Differentiation of Shitou Goose Myoblasts
Source: Animals (Basel). 2022 Oct 27;12(21):2956. doi: 10.3390/ani12212956 (PMC9658593; doi:10.3390/ani12212956)
Supplement: Supplementary file 1 [file animals-12-02956-s001.zip › Supplementary Table S1.pdf]

Supplementary information

Table S1. 43 differentially expressed genes with simultaneous differential splicing

DSGs VS DEGs							
**DNM1**	**CMYA5**	**GSG1L**	**GPC3**	**ABLIM1**	**ELN**	**LOC106033184**	**IL17REL**
**ADAM19**	**RUNX1T1**	**ZDHHC1**	**DMD**	**LOC106032910**	**QPCT**	**LOC106032265**	
**SLC9A9**	**CMKLR1**	**POSTN**	**KLHL5**	**MYO1C**	**ABLIM2**	**LOC106030353**	
**SORBS2**	**ZNF536**	**UNC5C**	**COL26A1**	**DHRS7C**	**P4HA2**	**DST**	
**KIF25**	**TPD52L1**	**TENM3**	**COL24A1**	**USP54**	**TNC**	**ADAMTS13**	
**SVIL**	**SMOC2**	**CELF2**	**SLC4A4**	**VLDLR**	**DAB2**	**PTPN3**	
