# Supplementary figures and images for: Transcriptome Sequencing Reveals Pathways Related to Proliferation and Differentiation of Shitou Goose Myoblasts
Source: Animals (Basel). 2022 Oct 27;12(21):2956. doi: 10.3390/ani12212956 (PMC9658593; doi:10.3390/ani12212956)

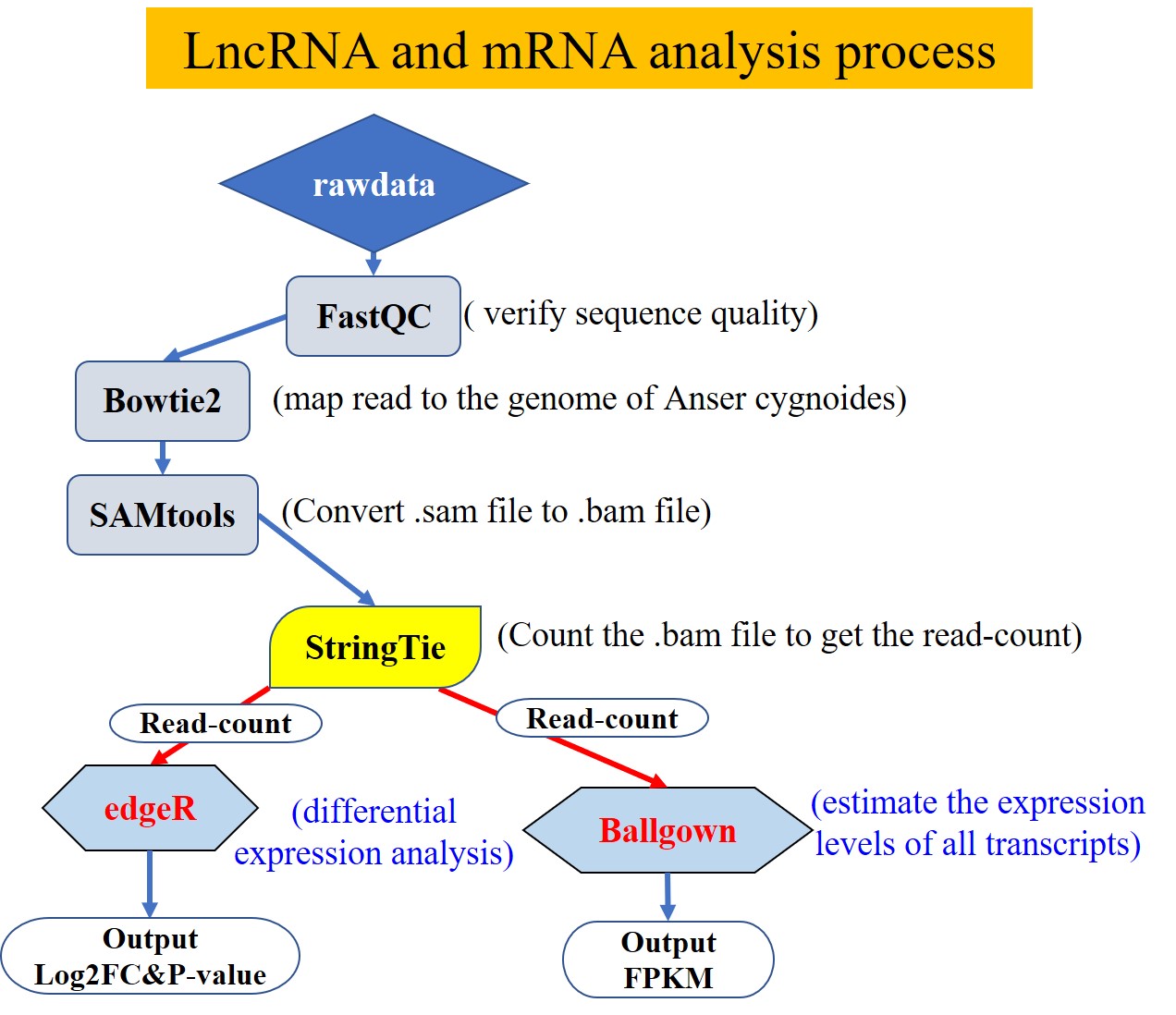

Supplement: Supplementary file 1 [file animals-12-02956-s001.zip › Supplementary Figure S1.jpg]

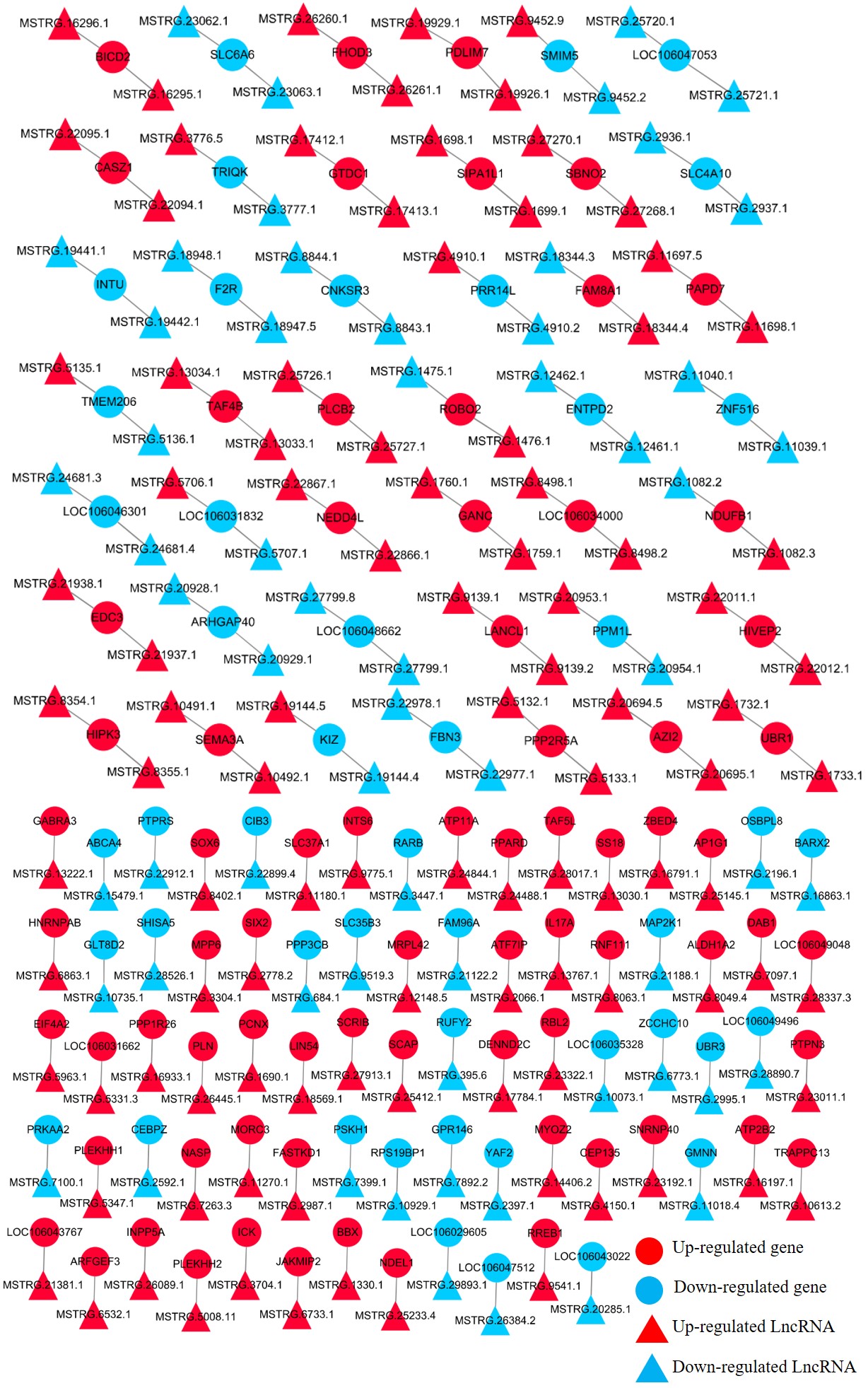

Supplement: Supplementary file 1 [file animals-12-02956-s001.zip › Supplementary Figure S2.jpg]
